# A simulation-based comparison of Boruta, LASSO, and Elastic Net for variable selection in logistic regression, with an ovarian cancer miRNA application

**DOI:** 10.1186/s12874-026-02939-5

**Published:** 2026-08-24

**Authors:** Reza Arabi Belaghi, Hulya Yurekli, Farzaneh Hamidi, Neda Gilani

**Affiliations:** 1 Unit of Applied Statistics and Mathematics, Department of Energy and Technology, Box 7032, Swedish University of Agricultural Sciences, Lennart Hjelms väg 9, Uppsala, SE-750 07 Sweden; 2 Department of Statistics, Yıldız Technical University, Esenler, Istanbul, 34220 Türkiye; 3 Department of Statistics and Epidemiology, Faculty of Health, Tabriz University of Medical Sciences, Tabriz, Iran

**Keywords:** Variable selection, High-dimensional data, Logistic regression, Boruta, LASSO, Elastic Net, Simulation study, miRNA

## Abstract

**Background:**

Variable selection is a central challenge in logistic regression, particularly in high-dimensional biomedical applications where correlated predictors and limited sample sizes complicate reliable identification of relevant variables. This study aims to systematically compare three widely used variable selection approaches - Boruta, LASSO, and Elastic Net - under a range of data-generating conditions and to illustrate their performance using an ovarian cancer miRNA dataset.

**Methods:**

We conducted a simulation study across 36 logistic regression scenarios varying in sample size, dimensionality, predictor correlation, and effect magnitude. Performance was evaluated using true-positive and false-positive selection rates. In addition, all three methods were applied to a real-world serum miRNA expression dataset, and discriminative performance was assessed using the area under the receiver operating characteristic curve (AUC).

**Results:**

Boruta, Elastic Net, and LASSO exhibited distinct variable selection behaviors across simulation scenarios. Boruta maintained strong true-positive recovery while controlling false positives in most settings, particularly when predictors were highly correlated. Elastic Net consistently achieved high sensitivity but produced comparatively large false-positive rates. LASSO showed the most conservative behavior, recovering fewer true predictors while maintaining low false-positive rates across nearly all scenarios. In the ovarian cancer miRNA application, all three methods achieved similarly strong test-set AUC performance, despite marked differences in the size of the selected biomarker panels.

**Conclusions:**

The results demonstrate clear trade-offs among the three methods. Boruta offers a favorable balance between sensitivity and specificity in highly correlated settings. Elastic Net prioritizes sensitivity at the cost of increased false discoveries, whereas LASSO provides stricter false-positive control with reduced sensitivity. These findings offer practical guidance for selecting variable selection methods in logistic regression, particularly for high-dimensional biomedical applications.

## Background

Variable selection, also known as feature selection, refers to the procedure of identifying the most meaningful predictors from a larger set of candidate variables. This process is particularly critical for improved performance and interpretability in high-dimensional settings [1], where the number of variables (*p*) approaches or surpasses the number of observations (*n*). High dimensional data can degrade classifier performance by increasing the likelihood of overfitting and computational burden [2, 3]. Effective variable selection in these contexts improves model interpretability, reduces the risk of overfitting, and enhances predictive performance [1, 3, 4].

Feature selection approaches are commonly categorized into filters, wrappers, and embedded methods, depending on how the selection mechanism is integrated with the modeling process [3, 5, 6]. Filter methods evaluate features based on intrinsic data characteristics (e.g., correlation, mutual information), wrapper methods assess subsets through model training and validation, and embedded methods integrate variable selection within the model-building process, enabling simultaneous optimization of feature selection and parameter estimation [7].

Among embedded approaches, regularization-based methods, especially the Least Absolute Shrinkage and Selection Operator (LASSO) [8], have received considerable attention. The LASSO imposes an $\ell _1$-norm penalty on regression coefficients, yielding sparse solutions that simultaneously perform variable selection and shrinkage [8, 9]. Owing to its ability to produce sparse models, LASSO has become increasingly attractive in modern data analysis [10]. However, LASSO has notable limitations: (1) when a group of variables exhibits high pairwise correlations, LASSO typically selects only one variable from the group; and (2) in situations where the number of predictors exceeds the number of observations (*p > n*), LASSO can select at most *n* variables due to constraints inherent in the convex optimization framework [11]. There are also no widely accepted guidelines on the applicability of LASSO in settings involving strong dependence among covariates [11].

The Elastic Net [10] was introduced as a hybrid regularization method that combines the $\ell _1$ penalty of LASSO with the $\ell _2$ penalty of ridge regression. While Elastic Net, similar to LASSO, performs simultaneous variable selection and continuous shrinkage, its additional grouped selection ability makes it more effective than LASSO in many correlated settings [10].

As an alternative to model-based selection techniques, the Boruta algorithm [12] offers a model-agnostic machine learning approach rooted in the principles of random forests. The Boruta algorithm introduces additional randomness into the model by generating shadow features, which are randomized copies of the original variables, and appends them to the dataset. A variable is considered relevant only if its importance score is significantly higher than the maximum importance observed among the shadow features [12].

Beyond regression-based methods, [13] systematically compared variable selection techniques for random forests using simulated and real omics data, and concluded that Boruta outperformed competing approaches by reliably identifying relevant variables and recommended its use alongside Vita for high-dimensional data sets. Similarly, [14] evaluated random-forest-based feature selection approaches and found that Boruta achieved the highest stability across different stringency settings, consistently identifying robust biomarkers. Because of its computational efficiency, Boruta is recommended for datasets with a large number of predictors [15].

Motivated by these findings, the present study systematically compares the performance of Boruta, LASSO, and Elastic Net across a set of simulation scenarios that vary in correlation structure, sample size, dimensionality (including the number of informative and noise variables), and coefficient magnitude. In addition to the simulation study, we applied Boruta, LASSO, and Elastic Net to a real-world microRNA (miRNA) expression dataset related to ovarian cancer [16], with the goal of identifying a subset of biomarkers predictive of case-control status. This combined approach, incorporating both simulation and real-data analysis, offers a comprehensive evaluation of each method’s relative strengths and limitations.

The paper is organized into five sections. Methods section details the baseline model, provides an overview of the Boruta, Elastic Net, and LASSO approaches, and describes the simulation framework. Results section summarizes the results obtained across the simulation scenarios. Application to real data: ovarian cancer microarray dataset section reports the application of the three methods to an ovarian cancer microarray dataset, and Discussion section concludes the study.

## Methods

### Baseline model: logistic regression

Logistic regression is one of the most widely used statistical models in medical and biomedical research for analyzing binary outcomes such as disease presence, treatment success, or diagnostic classification [17, 18]. In this study, data were generated from a binary logistic regression model for all simulation scenarios.

For subject *i = 1,… ,n*, let $Y_i \in \{0,1\}$ denote the binary outcome and $\textbf{x}_i = (x_{i1},\dots ,x_{ip})^\top$ the vector of predictors. The event probability $p_i = \Pr (Y_i = 1 \mid \textbf{x}_i)$ is modeled using the logit link

1 $$\begin{aligned} \text {logit}(p_i)& = \log \left( \frac{p_i}{1 - p_i} \right) \\&= \eta _i = \beta _0 + \sum \limits _{j=1}^{p} \beta _j x_{ij}, \end{aligned}$$

where $\beta _0$ is the intercept and $\beta _j$ are the regression coefficients for the predictors. Conditional on $\textbf{x}_i$, the outcome follows a Bernoulli distribution:

2 $$\begin{aligned} Y_i \mid \textbf{x}_i \sim \textrm{Bernoulli}(p_i). \end{aligned}$$

The (unpenalized) log-likelihood of the logistic regression model for a parameter vector $\boldsymbol{\beta } = (\beta _0,\beta _1,\dots ,\beta _p)^\top$ is

3 $$\begin{aligned} &\ell (\boldsymbol{\beta })= \sum \limits _{i=1}^{n} \left\{ Y_i \eta _i - \log \left( 1 + e^{\eta _i}\right) \right\} , \\&\quad \text {with } \eta _i = \beta _0 + \sum \limits _{j=1}^{p} \beta _j x_{ij}. \end{aligned}$$

All penalized regression methods considered below use this same log-likelihood $\ell (\boldsymbol{\beta })$ as the data-fitting component of their objective functions.

### Penalized regression methods

In penalized logistic regression, the regression coefficients are estimated by maximizing a penalized log-likelihood of the general form

4 $$\begin{aligned} \hat{\boldsymbol{\beta }} = \arg \max _{\boldsymbol{\beta }} \left\{ \ell (\boldsymbol{\beta }) - P_\lambda (\boldsymbol{\beta })\right\} , \end{aligned}$$

where $\ell (\boldsymbol{\beta })$ is given by (3), and $P_\lambda (\boldsymbol{\beta })$ is a penalty function depending on tuning parameter(s) *λ *. Throughout, the intercept $\beta _0$ is not penalized, and penalties are applied only to $\beta _1,\dots ,\beta _p$.

#### LASSO

The LASSO performs variable selection and regularization by imposing an $\ell _1$-norm penalty on the regression coefficients, which encourages sparsity in the model [8]. For logistic regression, the LASSO estimator is defined as

5 $$\begin{aligned} \hat{\boldsymbol{\beta }}^{\text {LASSO}} = \arg \max _{\boldsymbol{\beta }} \left\{ \ell (\boldsymbol{\beta }) - \lambda \sum \limits _{j=1}^{p} |\beta _j| \right\} , \end{aligned}$$

where *λ > 0* controls the strength of the penalty. Larger values of *λ * induce stronger shrinkage on the coefficients, resulting in some coefficients being reduced to zero and enabling variable selection.

#### Elastic Net

The Elastic Net is a regularization method that combines the $\ell _1$ (LASSO-type) and $\ell _2$ (ridge-type) penalties through a convex combination [10]. Its penalized log-likelihood objective is

6 $$\begin{aligned} \hat{\boldsymbol{\beta }}^{\text {EN}} = \arg \max _{\boldsymbol{\beta }} \left\{ \ell (\boldsymbol{\beta }) - \lambda _1 \sum \limits _{j=1}^{p} |\beta _j| - \lambda _2 \sum \limits _{j=1}^{p} \beta _j^2 \right\} , \end{aligned}$$

where $\lambda _1 \ge 0$ and $\lambda _2 \ge 0$ control the relative contributions of the $\ell _1$ and $\ell _2$ penalties, respectively. In practice, as implemented in glmnet, this is often reparameterized in terms of an overall penalty level *λ > 0* and a mixing parameter $\alpha \in [0,1]$, with

$$\begin{aligned} \lambda _1 = \alpha \lambda , \quad \lambda _2 = (1 - \alpha )\lambda . \end{aligned}$$

Both LASSO and Elastic Net models were fitted using the glmnet package [19] in *R* [20]. Penalty parameters were selected via 10-fold cross-validation, using the value of *λ * that minimized the cross-validated prediction error.

### Boruta algorithm

The Boruta algorithm [12] is a feature selection method designed to identify all relevant features in a dataset. It is built on the random forest classification algorithm, which is used to compute feature importance scores [21]. Unlike traditional feature selection techniques, Boruta extends the original dataset by adding randomized versions of each predictor, called *shadow variables*, created by permuting the values of the original variables. In each iteration, the importance of original features is compared to that of the shadow features. Based on this comparison, features are classified as relevant, irrelevant, or tentative. A step-by-step description of the algorithm is presented below. Boruta was implemented using the Boruta package [21] in *R* [20].

**Figure:**
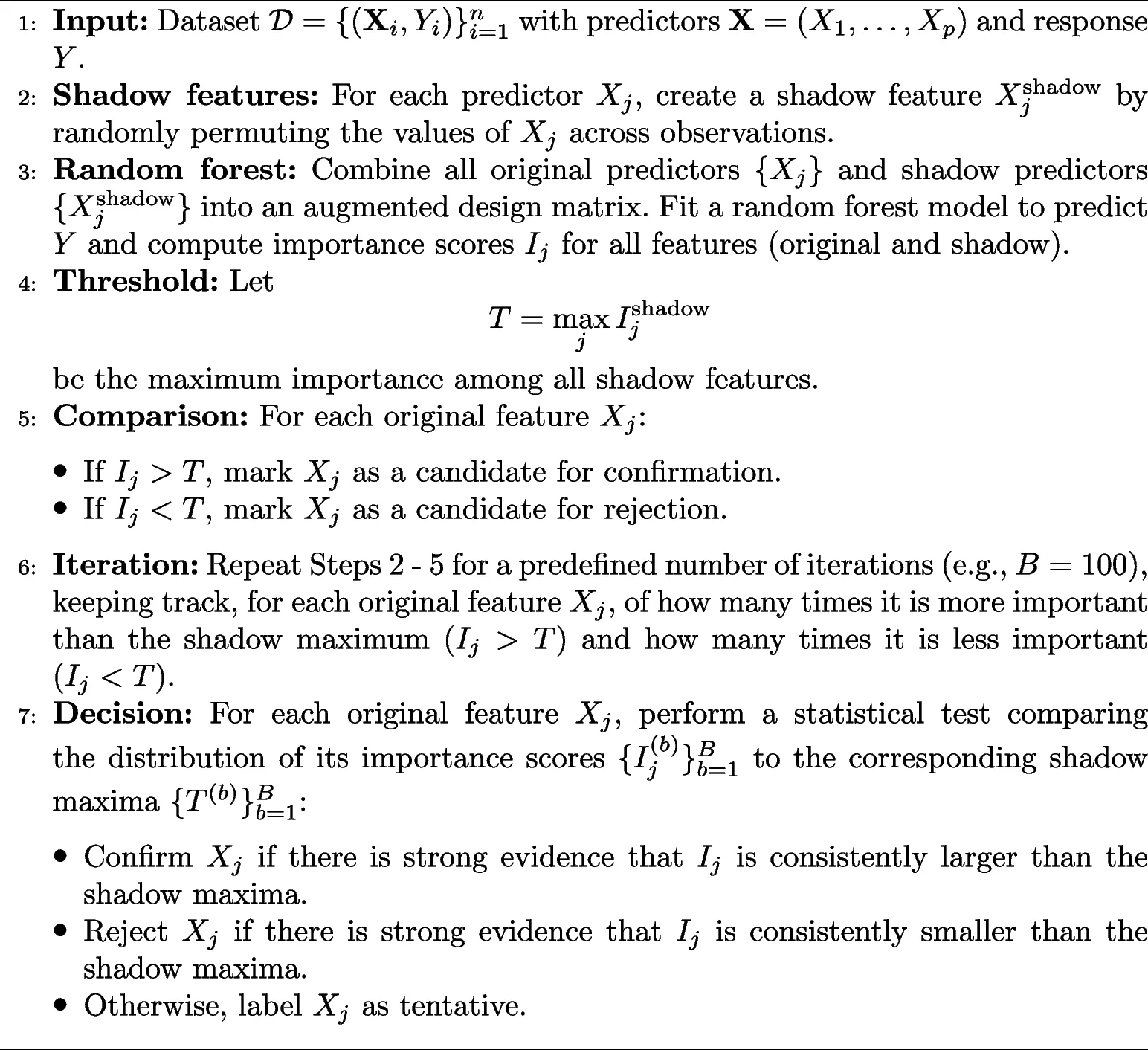
**Algorithm 1** Boruta feature selection using shadow features

### Simulation design

To evaluate the empirical performance of the Boruta, LASSO, and Elastic Net, we conducted a comprehensive set of simulations across a broad range of scenarios involving both high-dimensional and low-dimensional logistic regression settings. The primary objective of the simulation study was to assess variable selection accuracy rather than predictive performance; therefore, evaluation was based on true-positive and false-positive selection rates instead of test-set discrimination measures.

All datasets were generated from the logistic regression model described in Baseline model: logistic regression section. For each replication, the predictor vector $\textbf{x}_i = (x_{i1},\ldots ,x_{ip})^\top$ was drawn from a multivariate normal distribution with mean zero and a Toeplitz correlation structure,

$$\begin{aligned}& \textbf{x}_i \sim N_p \left( \textbf{0}, \boldsymbol{\Sigma }(\rho )\right) ,\\&\qquad \Sigma _{jk} = \rho ^{|j-k|}, \quad j,k = 1,\ldots ,p, \end{aligned}$$

with correlation parameter $\rho \in \{0.95, 0.75, 0.40\}$. Conditional on $\textbf{x}_i$, the binary outcome was generated via the inverse-logit transformation of the linear predictor

$$\begin{aligned}& \eta _i = \beta _0 + \sum \limits _{j=1}^p \beta _j x_{ij},\qquad \\&Y_i \mid \textbf{x}_i \sim \textrm{Bernoulli}\!\left( \frac{\exp (\eta _i)}{1+\exp (\eta _i)}\right) . \end{aligned}$$

The intercept was fixed at $\beta _0 = 0$. Each setting was replicated 1,000 times. Penalized regression models were tuned using 10-fold cross-validation.

We considered four main families of simulation scenarios (Scenarios 1 - 4). Within each family we varied sample size *n*, the dimensionality *p* (the number of truly informative predictors $p_1$ and the number of noise predictors $p_2 = p - p_1$), and the correlation parameter *ρ *, yielding 36 distinct combinations in total. In all scenarios, the true regression coefficient vector was $\boldsymbol{\beta } = (\beta _1,\ldots ,\beta _p)^\top$. We used three different coefficient patterns:

#### Pattern S1.

A set of predictors was assumed to have very strong effects for this pattern:

7 $$\begin{aligned} \beta _j^{(S1)} = \left\{ \begin{array}{ll} 10, & j = 1,\ldots ,p_1,\\ 0, & j = p_1+1,\ldots ,p. \end{array}\right. \end{aligned}$$

#### Pattern S2.

This pattern includes moderate and strong effects. Let $p_{11} = 3$ and $p_{12} = p_1 - p_{11} = 7$, with $p_1=10$ informative predictors:

8 $$\begin{aligned} \beta _j^{(S2)} = \left\{ \begin{array}{ll} 1.0, & j = 1,\ldots ,p_{11},\\ 0.5, & j = p_{11}+1,\ldots ,p_1,\\ 0, & j = p_1+1,\ldots ,p. \end{array}\right. \end{aligned}$$

#### Pattern S3.

In this pattern, the informative effects exhibit systematically decreasing magnitudes. Let $p_{11} = 10$, $p_{12} = 20$, and $p_{13} = p_1 - (p_{11}+p_{12}) = 20$, with $p_1=50$ informative predictors:

9 $$\begin{aligned} \beta _j^{(S3)} = \left\{ \begin{array}{ll} 0.10, & j = 1,\ldots ,p_{11},\\ 0.05, & j = p_{11}+1,\ldots ,p_{11}+p_{12},\\ 0.001, & j = p_{11}+p_{12}+1,\ldots ,p_1,\\ 0, & j = p_1+1,\ldots ,p. \end{array}\right. \end{aligned}$$

The combinations of $(n,~p,~p_1,~p_2,~\rho )$ used in each scenario family, along with the corresponding coefficient patterns, are summarized in Table 1.

**Table 1:** Overview of simulation scenarios for evaluating variable selection methods. For each row, three correlation settings were used: $\rho \in \{0.95, 0.75, 0.40\}$

Scenario family	Dimensionality	*n*	*p*_1_	*p*_2_	*p*	Coefficient pattern
1.1A-C	High-dimensional	50	10	50	60	Pattern S1 (7)
1.2A-C	High-dimensional	50	10	200	210	Pattern S1 (7)
1.3A-C	High-dimensional	50	10	500	510	Pattern S1 (7)
2.1A-C	High-dimensional	50	10	100	110	Pattern S2 (8)
2.2A-C	High-dimensional	50	10	200	210	Pattern S2 (8)
2.3A-C	High-dimensional	50	10	500	510	Pattern S2 (8)
3.1A-C	High-dimensional	50	50	500	550	Pattern S3 (9)
3.2A-C	High-dimensional	50	50	1000	1050	Pattern S3 (9)
3.3A-C	High-dimensional	50	50	1500	1550	Pattern S3 (9)
4.1A–C	Low-dimensional	100	10	10	20	Pattern S2 (8)
4.2A–C	Low-dimensional	200	10	10	20	Pattern S2 (8)
4.3A–C	Low-dimensional	500	10	10	20	Pattern S2 (8)

## Results

**Fig. 1:**
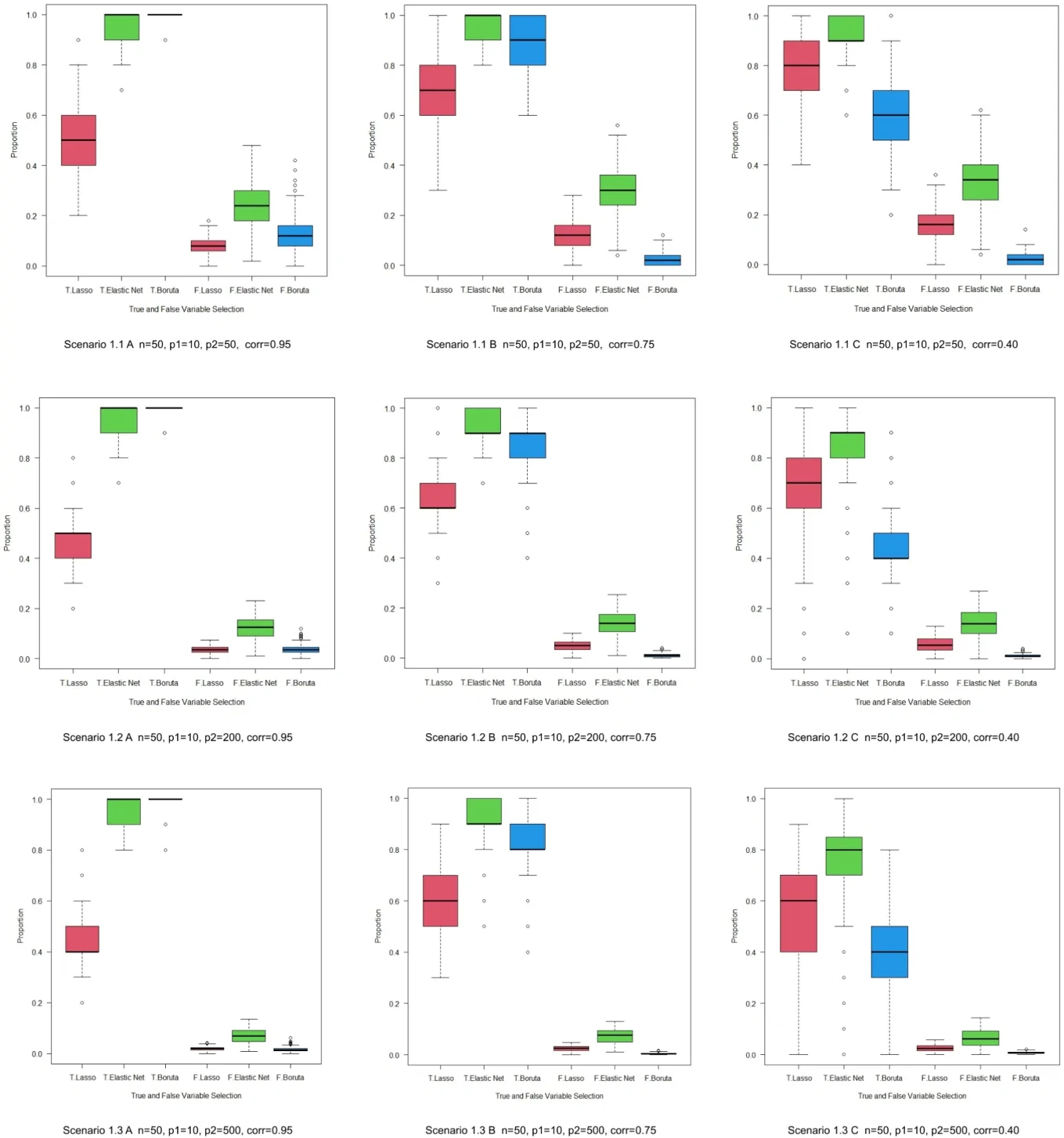
Distributions of true and false positives for LASSO, Elastic Net, and Boruta in Scenario 1

**Fig. 2:**
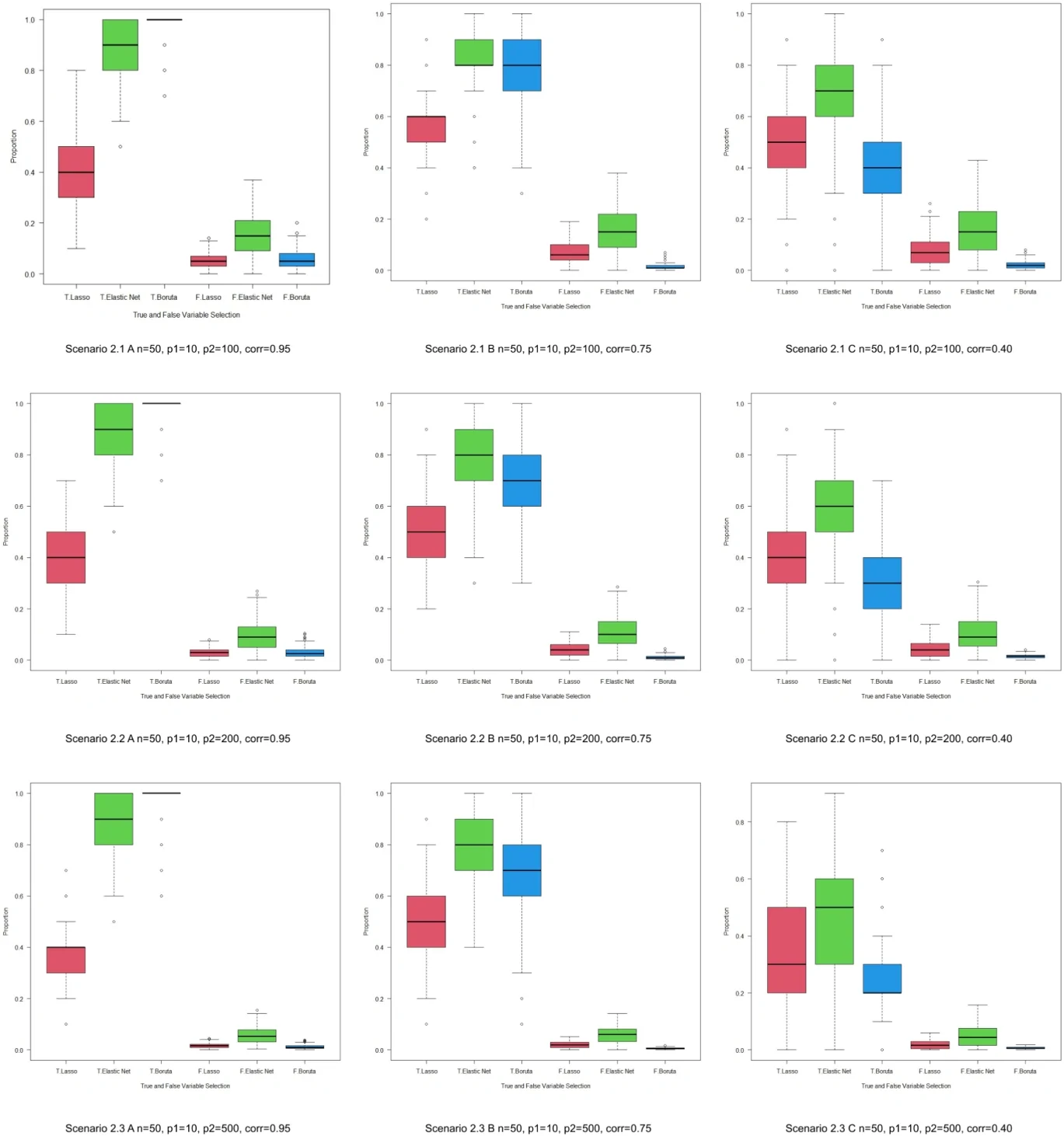
Distributions of true and false positives for LASSO, Elastic Net, and Boruta in Scenario 2. *Note.* y-axis scales differ across panels; therefore, cross-panel comparisons should be interpreted using the reported distributions and medians rather than absolute vertical distances

**Fig. 3:**
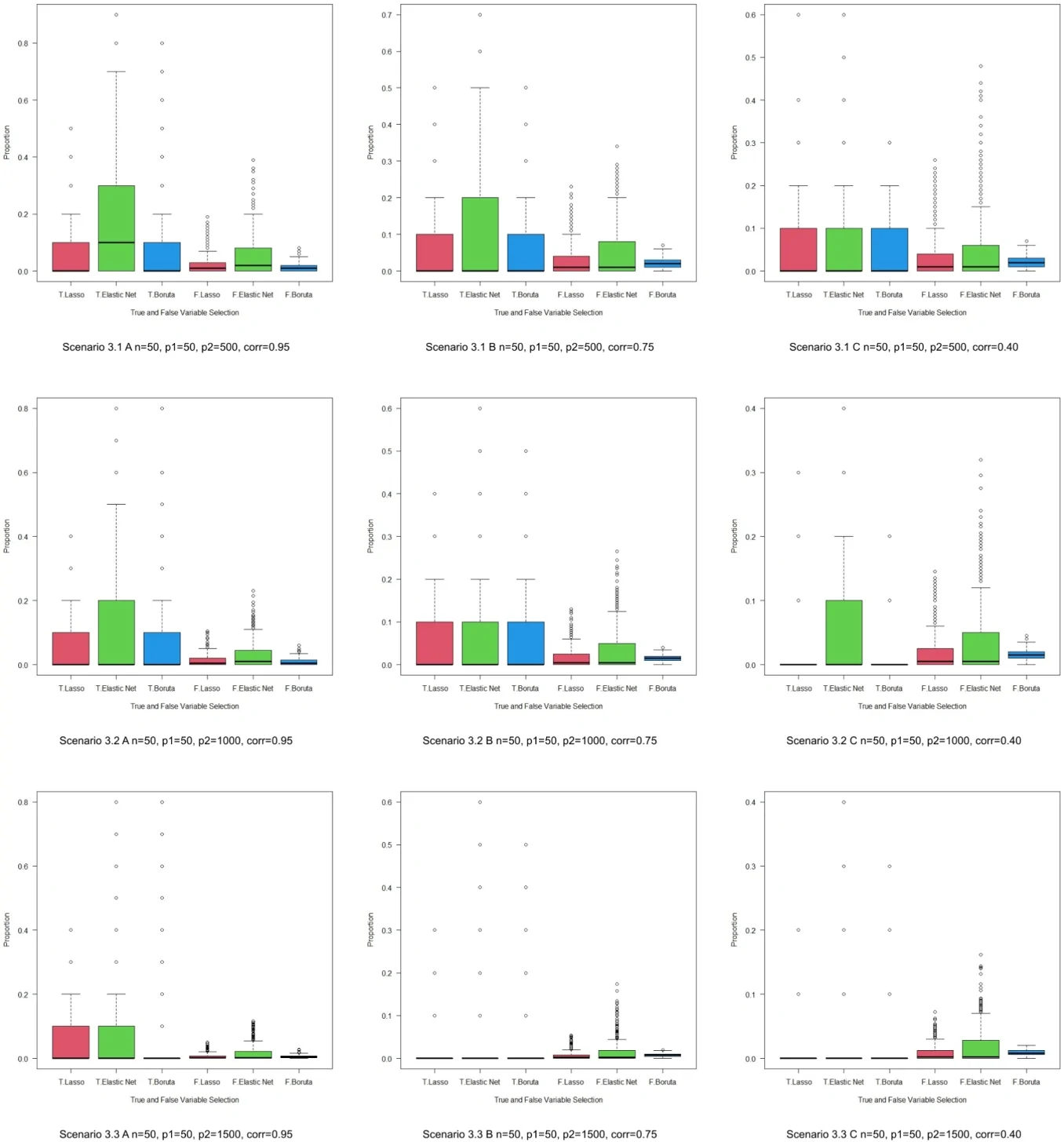
Distributions of true and false positives for LASSO, Elastic Net, and Boruta in Scenario 3. *Note.* y-axis scales differ across panels; therefore, cross-panel comparisons should be interpreted using the reported distributions and medians rather than absolute vertical distances

**Fig. 4:**
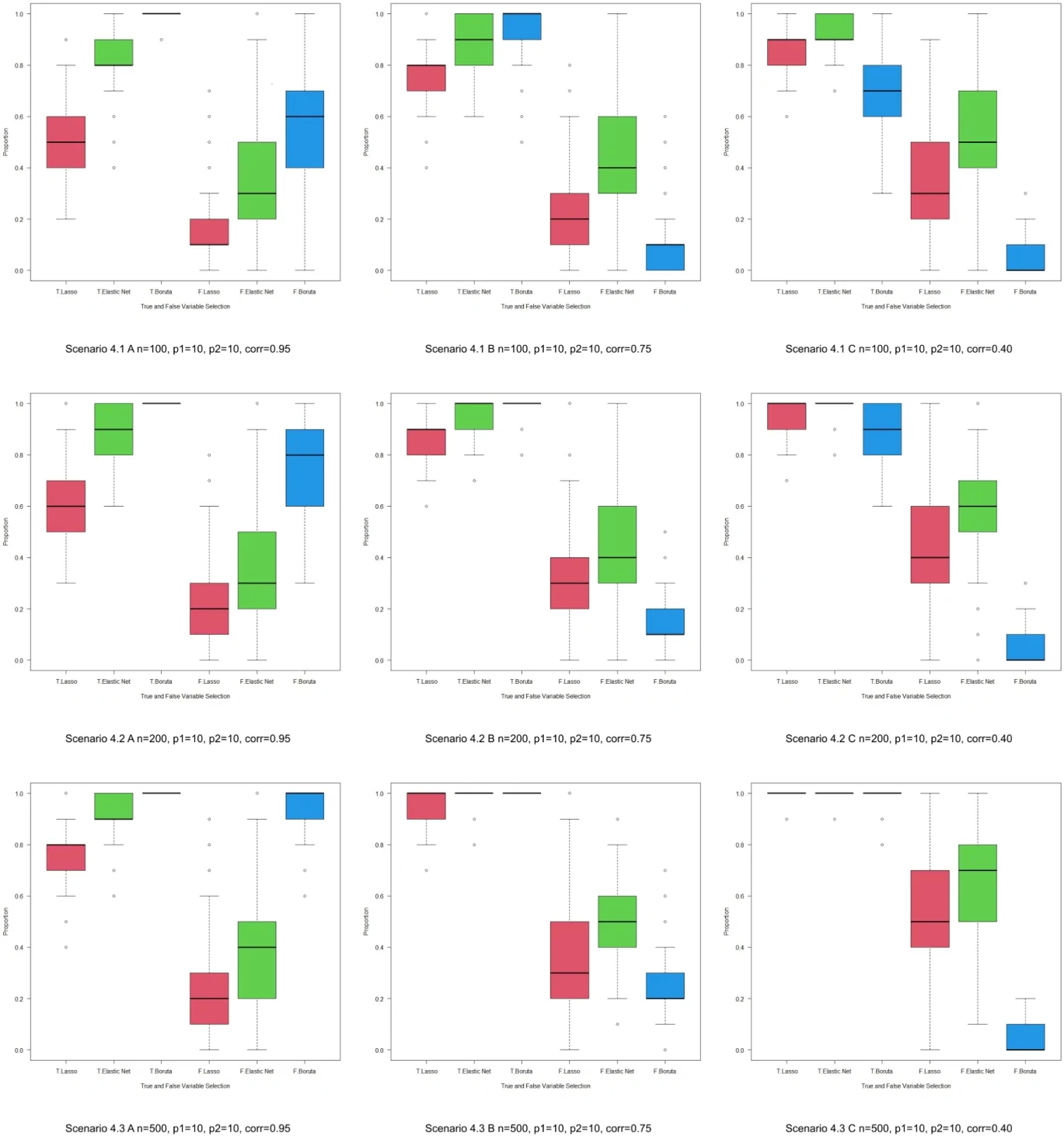
Distributions of true and false positives for LASSO, Elastic Net, and Boruta in Scenario 4

For each simulation scenario, we computed the true positive (TP) rates, defined as the proportion of informative predictors correctly identified, and the false positive (FP) rates, defined as the proportion of noise predictors incorrectly selected. The simulation study focused on variable selection performance; therefore, evaluation was based on the TP and FP rates. The distributions of TP and FP rates across all 36 simulation conditions for LASSO, Elastic Net, and Boruta are summarized in Figs. 1, 2, 3 and 4. Each figure is arranged in a *3× 3* grid. Across all figures, correlation among predictors decreases from left to right (*ρ = 0.95,~0.75,~0.40*), whereas dimensionality or sample size increases from top to bottom, depending on the scenario.

### Scenario 1. High-dimensional setting with very strong effects: *n = 50*, $p_1 = 10$, $p_2 \in \{50, 200, 500\}$

Scenario 1 (Scenarios 1.1A - 1.3C) corresponds to a high-dimensional configuration with *n=50*, a relatively small set of informative predictors ($p_1=10$), and increasing numbers of noise variables ($p_2 = 50,~200,~500$). Some of the true effects were set to a large value (*β = 10*), and correlation among predictors ranged from *ρ =0.40* to *ρ =0.95*.

Boruta recovers a large proportion of the true predictors when correlation is strong (*ρ = 0.95*), with TP rates close to one across all values of $p_2$ (Fig. 1). Its TP rates remain high at *ρ = 0.75*, but drop substantially when correlation decreases to *ρ = 0.40*. Under *ρ = 0.40*, TP rates also decline as $p_2$ increases. Boruta’s FP rates remain low and close to zero in nearly all settings.

Elastic Net yields consistently high TP rates, with values close to one, across all correlation levels and dimensionalities. However, it also produces the highest FP rates among the three methods. Although Elastic Net’s FP rates tend to decrease as $p_2$ increases, they remain substantially higher than those of both Boruta and LASSO in every panel.

LASSO’s TP rates are lower than both Boruta and Elastic Net at correlation levels *ρ = 0.95* and *ρ = 0.75*, and are lower than Elastic Net at *ρ = 0.40*. Its FP rates, however, remain low across all scenarios.

Overall in Scenario 1, Boruta provides strong recovery of informative predictors under strong correlation, with mostly low FP rates; Elastic Net achieves high TP rates but at the cost of high FP rates; and LASSO selects fewer predictors while maintaining relatively low FP rates.

### Scenario 2. High-dimensional setting with moderate and strong effects: *n = 50*, $p_1 = 10$, $p_2 \in \{100, 200, 500\}$

Scenario 2 (Scenarios 2.1A - 2.3C) considers settings with informative predictors ($p_1$) equal to 10, $p_2$ is from 100 to 500, some non-zero coefficients set to *β = 0.5*, and others to *β = 1.0*. Correlation among predictors varies from *ρ = 0.40* to *ρ = 0.95*.

Boruta achieves high TP rates under strong correlation (*ρ = 0.95*) (Fig. 2). As correlation decreases to *ρ = 0.75*, Boruta’s TP rates remain relatively high but show some decrease. At *ρ = 0.40*, its TP rates drop more markedly, particularly when $p_2$ is large. Boruta’s FP rates are low across most conditions.

Elastic Net yields high TP rates across nearly all combinations of $p_2$ and *ρ *, with TPs close to one at *ρ = 0.95* and *ρ = 0.75*. However, this performance is accompanied by higher FP rates than those observed for Boruta and LASSO. These FP rates tend to decrease as $p_2$ grows.

LASSO’s TP rates were lower than Boruta’s and Elastic Net’s at *ρ = 0.95* and *ρ = 0.75*, and were lower than Elastic Net’s at *ρ = 0.40*. Its FP rates were low across all panels.

In summary, Scenario 2 shows that Boruta maintains high TP rates at *ρ = 0.95* with low FP rates. Elastic Net achieves high sensitivity at *ρ = 0.95* with some decrease at *ρ = 0.75* but selects more noise variables. LASSO selects a smaller subset of predictors with low FP rates.

### Scenario 3. High-dimensional setting with weak effects: *n = 50*, $p_1 = 50$, $p_2 \in \{500, 1000, 1500\}$

Scenario 3 (Scenarios 3.1A - 3.3C) reflects a high dimensional configuration with informative predictors ($p_1$) and sample size (*n*) equal to 50, and the number of noise variables is ($p_2$) up to 1500. The informative effects take values of 0.10, 0.05, and 0.001, and correlation among predictors ranged from *ρ = 0.40* to *ρ = 0.95*. Scenario 3 was designed as a deliberately challenging weak-signal setting intended to stress-test the variable selection methods under extremely low effect sizes in high-dimensional data, rather than to represent a typical applied analysis scenario.

All three methods struggle to recover a substantial proportion of the true predictors (Fig. 3). Although performance improves slightly when the correlation is strong (*ρ = 0.95*), the TP rates remain low across all methods. As correlation decreases to *ρ = 0.75* and *ρ = 0.40*, recovery becomes even more difficult, with TP rates dropping further across all methods. As $p_2$ grows from 500 to 1500, TP rates for all three methods tend to move towards zero. Even though Elastic Net typically recovers more predictors than Boruta and LASSO, the overall ability to identify true signals is poor across all panels in this scenario family. Regarding FPs, all three methods maintain relatively low FP rates. LASSO and Boruta keep FPs close to zero across all settings, while Elastic Net shows a little higher FP rates, though these are still modest on the 0 - 1 scale and tend to be smaller at larger $p_2$ values. The poor recovery observed in this scenario should therefore be interpreted as evidence of the limits of all three methods under extremely weak signal conditions, rather than as a failure in settings where stronger effects are expected.

### Scenario 4. Low-dimensional setting with moderate and strong effects: $n \in \{100, 200, 500\}$, $p_1 = 10$, $p_2 = 10$

Scenario 4 (Scenarios 4.1A - 4.3C) examines a low-dimensional configuration with an equal number of informative and noise predictors ($p_1=p_2=10$) with sample sizes ranging from *n=100* to *n=500*. Some of the non-zero coefficients were set to *β =0.5* and *β =1.0*, and correlation among predictors ranged from *ρ = 0.40* to *ρ = 0.95*.

Boruta achieves high TP rates in all scenarios with values often close to one, except Scenario 4.1C (Fig. 4). Its FP rates remain low when *ρ = 0.40* and generally low at *ρ = 0.75*, but increase under strong correlation (*ρ = 0.95*). At *ρ = 0.75* and *ρ = 0.95*, FP rates also tend to rise as *n* increases. One possible explanation is that, as the sample size increases, Boruta becomes more sensitive not only to truly informative predictors but also to predictors that are correlated with them. Because Boruta is an all-relevant, importance-based selection method, larger samples may produce more stable importance estimates, allowing correlated noise variables to exceed the shadow-feature threshold more consistently. Under our simulation definition, such variables are counted as FPs even though they may carry indirect information through their association with the true predictors.

Elastic Net tends to produce high TP rates with values close to one across all *n* and *ρ * combinations in this scenario. However, its FP rates are usually high.

LASSO tends to yield lower TP rates than Boruta and Elastic Net when *ρ = 0.95* and *ρ = 0.75*. However, its sensitivity increases as the sample size grows. LASSO’s FP rates are lower than both Elastic Net’s and Boruta’s when *ρ = 0.95*, and lower than Elastic Net’s when *ρ = 0.75* and *ρ = 0.40*.

Overall, increasing the sample size improves TP recovery for all three methods in this scenario. When *ρ = 0.95*, Boruta has the highest sensitivity but with the highest FP rates. When *ρ = 0.75*, Boruta has the highest TP rates with the lowest FPs. Elastic Net generally achieves high TP rates but with usually high FP rates. LASSO provides the lowest sensitivity with the lowest FP rates at *ρ = 0.95*.

Across all four scenarios, Boruta consistently demonstrates robust TP recovery at *ρ = 0.95* except for the ultra-high dimensional setting in Scenario 3. The FP rates of Boruta are generally low in high-dimensional settings. Boruta’s sensitivity gets higher with the higher correlation levels in Scenarios 1 and 2. Although lower predictor correlation may seem to simplify variable selection in regression settings, Boruta relies on repeated random-forest importance comparisons against shadow variables rather than on coefficient estimation alone. In the present simulations, strong correlation among informative predictors may have created a more stable shared relevance pattern, which improved Boruta’s ability to distinguish true signals from shadow features. When correlation was weaker, this collective structure was reduced, and Boruta’s TP recovery declined. This interpretation is specific to the simulation design considered here and should not be taken to imply that stronger correlation universally improves feature selection. Elastic Net exhibits high TP rates overall, often close to one across combinations of *p*, *n*, and *ρ *, but this advantage comes at the cost of noticeably higher FP rates. LASSO consistently yields low TP rates, but compensates with the low FP rates across most scenarios. Taken together, the simulation results highlight a clear trade-off: Boruta provides a strong balance of sensitivity and specificity under strong or moderate effects, Elastic Net maximizes sensitivity at the expense of higher false positives, and LASSO offers strict FP control while recovering fewer true predictors.

## Application to real data: ovarian cancer microarray dataset

miRNAs are small non-coding RNA sequences that modulate gene expression after transcription and contribute to essential cellular functions, including apoptosis, proliferation, and metastasis [22]. Their dysregulation has been associated with oncogenic transformation and tumor suppression, making them promising candidates for cancer diagnostics and prognostics [23].

To further assess the practical utility of Boruta in comparison with LASSO and Elastic Net, we applied all three methods to the GSE106817 dataset [16], which contains serum miRNA expression profiles from ovarian malignancy patients. For the present analysis, we restricted the data to the binary comparison of ovarian cancer versus non-cancer controls. The resulting subset included 3,079 samples, consisting of 320 ovarian cancer cases and 2,759 non-cancer controls, with 2,565 miRNA features and no missing expression values. Because the case-control comparison is highly imbalanced, we did not rely on overall accuracy alone. Instead, we used a stratified 70%/30% train/test split, applied random downsampling only within the training set, and evaluated final performance on the untouched test set. Accordingly, class imbalance was handled through training-set downsampling rather than class weighting, and evaluation emphasized the area under the receiver operating characteristic curve (AUC), sensitivity, specificity, accuracy, and balanced accuracy.

We first examined the correlation structure and marginal distributional characteristics of the raw miRNA predictors. Across all 2,565 miRNAs, the mean pairwise correlation was 0.155, the median pairwise correlation was 0.157, the mean absolute correlation was 0.196, the median absolute correlation was 0.174, the 95th percentile of the absolute correlations was 0.446, and the maximum absolute correlation was 0.965. Although the average pairwise correlation was modest, the upper tail of the correlation distribution indicated that some miRNA pairs were moderately to strongly correlated.

We also assessed the marginal distributions of the raw miRNA predictors across all 2,565 miRNAs. The mean skewness was *-0.289*, whereas the median skewness was *-0.017*. In total, 67.2% of miRNAs had absolute skewness greater than 0.5, 38.3% exceeded 1.0, and 10.7% exceeded 2.0, with skewness values ranging from *-6.136* to 1.967. These findings indicated that the raw miRNA predictors were not approximately normal. Therefore, we applied a rank-based inverse normal transformation separately to each miRNA so that the marginal predictor distributions were approximately Gaussian, and then performed the real-data analysis using the normalized predictors.

**Fig. 5:**
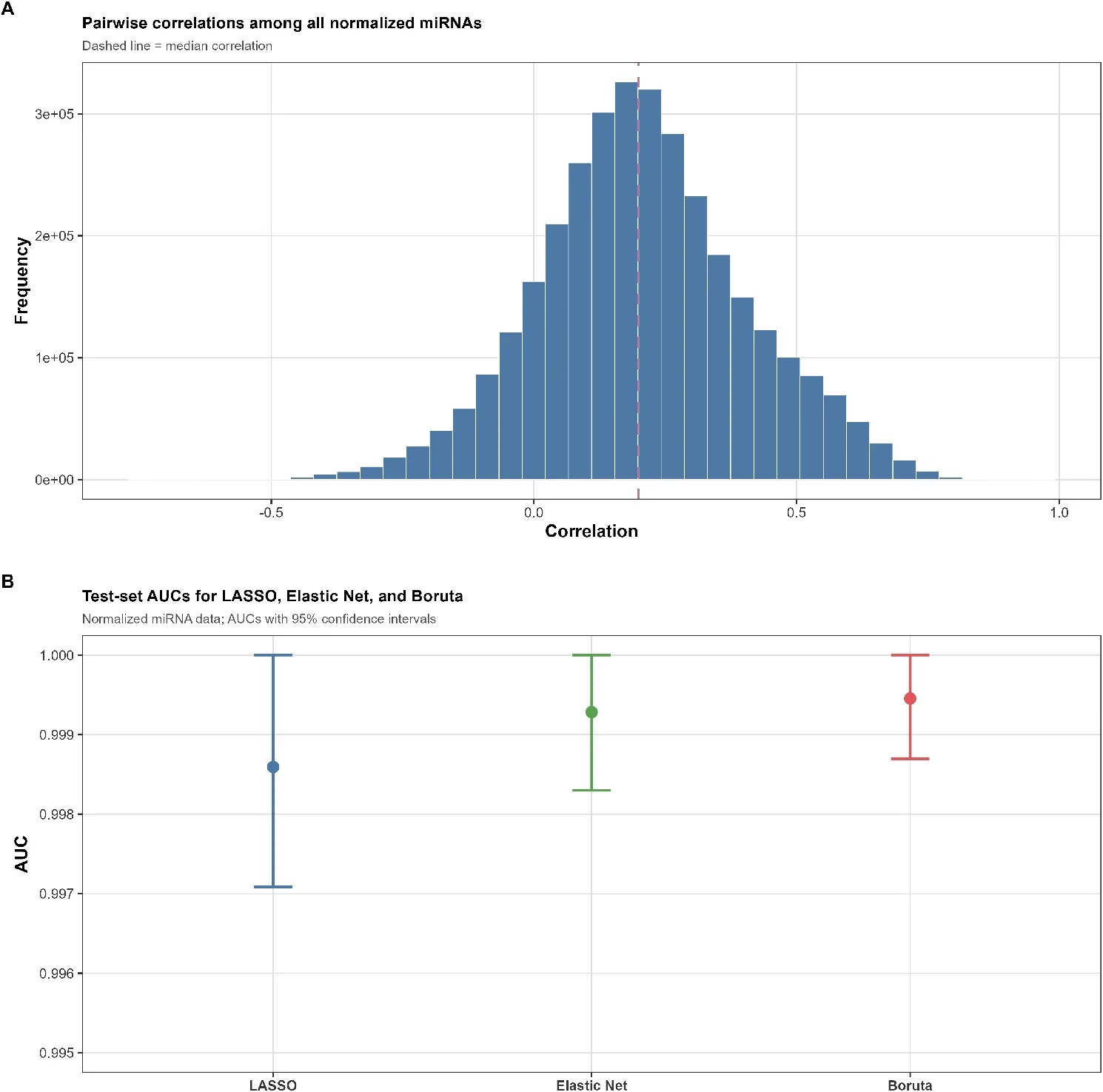
Correlation structure and test-set predictive performance in the normalized GSE106817 ovarian cancer dataset. **A** Histogram of pairwise correlations among all normalized miRNAs; the dashed vertical line marks the median correlation. **B** Test-set AUCs with 95% confidence intervals for LASSO, Elastic Net, and Boruta after normalization of the miRNA predictors

The normalization substantially reduced skewness across the predictors. After transformation, the mean skewness was approximately 0.000 and the median skewness was approximately 0.000, and no normalized miRNA had absolute skewness greater than 0.5 (Fig. 5A). The normalized predictors exhibited a mean pairwise correlation of 0.206, a median pairwise correlation of 0.199, a mean absolute correlation of 0.235, a 95th percentile absolute correlation of 0.555, and a maximum absolute correlation of 0.970. Thus, normalization made the marginal distributions more comparable to the simulation assumptions, while preserving a non-negligible correlation structure among predictors.

LASSO, Elastic Net, and Boruta were then applied to the normalized training data for variable selection. LASSO selected 19 miRNAs, Elastic Net selected 51 miRNAs, and Boruta selected 135 miRNAs. This ordering was consistent with the broader simulation results, where LASSO generally behaved more conservatively, Elastic Net selected larger sets of predictors, and Boruta often identified a broader set of relevant variables under correlated settings. To summarize the selected features, miRNAs were ranked within each method using method-specific importance measures: absolute nonzero coefficient magnitude for LASSO and Elastic Net, and median Boruta importance for Boruta. Because these measures are method-specific, ranks should be interpreted within methods rather than compared numerically across methods. The top-ranked markers showed partial agreement across methods. MIMAT0005880, MIMAT0022259, MIMAT0005866, and MIMAT0027500 appeared among the five highest-ranked miRNAs for both LASSO and Elastic Net, while MIMAT0022259, MIMAT0005880, and MIMAT0027500 were also among the five highest-ranked Boruta-selected miRNAs (Table 2). The full ranked list of selected miRNAs are provided in Appendix: Table 3.

**Table 2:** Top 19 ranked selected miRNAs by LASSO, Elastic Net, and Boruta

Rank	LASSO	Elastic Net	Boruta
1	**MIMAT0005880**	**MIMAT0005880**	**MIMAT0022259**
2	**MIMAT0005866**	**MIMAT0022259**	**MIMAT0005880**
3	**MIMAT0022259**	**MIMAT0005866**	**MIMAT0005792**
4	**MIMAT0027500**	**MIMAT0004952**	**MIMAT0027500**
5	**MIMAT0022943**	**MIMAT0027500**	MIMAT0027468
6	**MIMAT0027462**	**MIMAT0021082**	MIMAT0015064
7	**MIMAT0021082**	**MIMAT0019852**	MIMAT0027504
8	**MIMAT0027496**	**MIMAT0005929**	MIMAT0005582
9	**MIMAT0019071**	**MIMAT0022943**	MIMAT0031000
10	**MIMAT0004952**	**MIMAT0027462**	**MIMAT0005866**
11	**MIMAT0021127**	MIMAT0027468	**MIMAT0022943**
12	**MIMAT0019947**	**MIMAT0027496**	**MIMAT0019071**
13	**MIMAT0019852**	**MIMAT0005792**	MIMAT0000510
14	**MIMAT0005929**	**MIMAT0019947**	**MIMAT0019947**
15	**MIMAT0005792**	**MIMAT0019229**	MIMAT0018085
16	**MIMAT0005922**	MIMAT0030998	MIMAT0019757
17	MIMAT0031119	MIMAT0018085	MIMAT0005951
18	**MIMAT0019229**	MIMAT0031119	MIMAT0003326
19	**MIMAT0028117**	**MIMAT0019071**	MIMAT0027565

Bold entries indicate miRNAs selected by all three methods

To evaluate predictive discrimination, we focused on test-set AUCs computed from the predicted probabilities of the final classifiers. All three approaches achieved excellent test-set discrimination on the normalized data. The test-set AUC was 0.9986 for LASSO, 0.9993 for Elastic Net, and 0.9995 for Boruta (Fig. 5B). Balanced accuracies were also high, at 0.9773 for LASSO, 0.9798 for Elastic Net, and 0.9792 for Boruta. Elastic Net and Boruta achieved perfect sensitivity (1.0000), while LASSO achieved a sensitivity of 0.9880. Specificity was slightly higher for LASSO (0.9667) than for Elastic Net (0.9595) and Boruta (0.9583). Accuracy was similar across methods, with values of 0.9686 for LASSO, 0.9632 for Elastic Net, and 0.9621 for Boruta. These results indicate that all three methods performed well on the minority ovarian cancer class, while LASSO showed slightly better specificity for the non-cancer controls.

Overall, the ovarian cancer miRNA dataset represents a high-dimensional biomarker setting in which some predictors were moderately to strongly correlated. This correlation structure remained after marginal normalization, making the application comparable to the correlated simulation settings. The normalized real-data analysis showed that all three methods achieved excellent test-set discrimination, while LASSO yielded the most parsimonious panel and Elastic Net and Boruta selected broader sets of biomarkers. Taken together, these findings were broadly consistent with the simulation results: LASSO produced the most parsimonious biomarker panel, whereas Elastic Net and Boruta selected broader sets of predictors while maintaining excellent test-set discrimination.

## Discussion

This study conducted a comprehensive simulation-based comparison of Boruta, Elastic Net, and LASSO across 36 logistic regression scenarios that varied in dimensionality, correlation structure, sample size, and effect magnitude. The findings reveal clear and consistent performance patterns that highlight the strengths and limitations of each method under different simulation conditions.

Across all scenarios, Boruta demonstrated strong performance in recovering true predictors when effect sizes were moderate or strong and correlation among predictors was high (*ρ = 0.95*). Its TP rates remained relatively stable in many configurations, and its FP rates were generally low. Boruta offered a favorable balance between sensitivity and specificity, particularly in Scenarios 1, 2, and the low-dimensional Scenario 4, indicating that it is well suited for most of the settings.

Elastic Net consistently achieved high TP rates among the three methods. However, these gains in sensitivity came at the expense of noticeably elevated FP rates. Elastic Net therefore provides a powerful but more liberal variable selection strategy, excelling in scenarios where identifying as many true predictors as possible is prioritized over strict FP control.

LASSO, in contrast, offered the most conservative behavior across all simulation scenarios. It consistently produced low TP rates, but maintained low FP rates in nearly every setting. These results indicate that LASSO remains an effective choice when parsimony and strict error control are the primary goals.

The real-data application involving serum miRNA profiling of ovarian cancer patients was broadly consistent with these conclusions. After applying a rank-based inverse normal transformation to the miRNA predictors, all three methods achieved excellent test-set discrimination, with AUCs of 0.9986 for LASSO, 0.9993 for Elastic Net, and 0.9995 for Boruta. Despite substantial differences in the number of selected miRNAs, the three approaches produced similarly strong discrimination. LASSO yielded the most parsimonious panel, whereas Elastic Net and Boruta selected broader sets of biomarkers, consistent with the simulation-based trade-off between conservative selection, grouped selection, and all-relevant selection in correlated settings.

Future research could extend the present study in several ways. One notable finding was that when effect sizes were weak, all algorithms struggled to correctly identify the truly significant variables. Future studies could address this limitation by exploring larger sample sizes, alternative modeling strategies, or more informative prior structures. Second, although the current simulation framework was designed primarily to evaluate variable selection accuracy, additional simulation studies could incorporate independent external test sets to assess predictive discrimination measures such as AUC alongside TP and FP selection rates. Third, future work could examine low-dimensional settings with extremely weak effects in order to better disentangle the impact of dimensionality from that of near-noise signal strength.

## Conclusion

This study presented a comprehensive simulation-based comparison of Boruta, Elastic Net, and LASSO across 36 logistic regression scenarios varying in dimensionality, correlation structure, sample size, and effect magnitude, complemented by a real-data application using serum miRNA profiles from ovarian cancer patients. Taken together, the simulation and empirical findings reveal a clear trade-off among the three methods. Boruta consistently achieved a favorable balance between sensitivity and specificity, under strong predictor correlation. Elastic Net demonstrated high sensitivity but tended to select a larger number of false positives, while LASSO exhibited conservative selection behavior, effectively controlling false positives at the expense of reduced sensitivity. These results highlight that method selection should be guided by the analytic objective - whether prioritizing comprehensive signal detection, strict control of false discoveries, or a balanced compromise between the two. The real-data application further corroborated these patterns, reinforcing the practical relevance of the simulation findings for logistic regression analyses in high-dimensional settings.

## Data Availability

Availability of data and materials: The real-data application in this study used a publicly available serum miRNA expression dataset from the Gene Expression Omnibus, accession number GSE106817, available at https://www.ncbi.nlm.nih.gov/geo/query/acc.cgi?acc=GSE106817. The simulated datasets and accompanying R scripts used to perform the analyses are available from the corresponding author upon reasonable request.

## Appendix

**Table 3:** Full ranked selected miRNA lists by LASSO, Elastic Net, and Boruta

Rank	LASSO	Elastic Net	Boruta
1	**MIMAT0005880**	**MIMAT0005880**	**MIMAT0022259**
2	**MIMAT0005866**	**MIMAT0022259**	**MIMAT0005880**
3	**MIMAT0022259**	**MIMAT0005866**	**MIMAT0005792**
4	**MIMAT0027500**	**MIMAT0004952**	**MIMAT0027500**
5	**MIMAT0022943**	**MIMAT0027500**	MIMAT0027468
6	**MIMAT0027462**	**MIMAT0021082**	MIMAT0015064
7	**MIMAT0021082**	**MIMAT0019852**	MIMAT0027504
8	**MIMAT0027496**	**MIMAT0005929**	MIMAT0005582
9	**MIMAT0019071**	**MIMAT0022943**	MIMAT0031000
10	**MIMAT0004952**	**MIMAT0027462**	**MIMAT0005866**
11	**MIMAT0021127**	MIMAT0027468	**MIMAT0022943**
12	**MIMAT0019947**	**MIMAT0027496**	**MIMAT0019071**
13	**MIMAT0019852**	**MIMAT0005792**	MIMAT0000510
14	**MIMAT0005929**	**MIMAT0019947**	**MIMAT0019947**
15	**MIMAT0005792**	**MIMAT0019229**	MIMAT0018085
16	**MIMAT0005922**	MIMAT0030998	MIMAT0019757
17	MIMAT0031119	MIMAT0018085	MIMAT0005951
18	**MIMAT0019229**	MIMAT0031119	MIMAT0003326
19	**MIMAT0028117**	**MIMAT0019071**	MIMAT0027565
20		MIMAT0015064	MIMAT0000690
21		**MIMAT0021127**	MIMAT0016879
22		MIMAT0022742	**MIMAT0019229**
23		MIMAT0031000	MIMAT0026477
24		MIMAT0027430	**MIMAT0019852**
25		**MIMAT0005922**	MIMAT0019791
26		**MIMAT0028117**	MIMAT0022742
27		MIMAT0027670	MIMAT0019691
28		MIMAT0019957	MIMAT0015079
29		MIMAT0027510	**MIMAT0021127**
30		MIMAT0027472	**MIMAT0027462**
31		MIMAT0027504	MIMAT0019778
32		MIMAT0023694	**MIMAT0021082**
33		MIMAT0019791	MIMAT0019957
34		MIMAT0022947	MIMAT0015072
35		MIMAT0030420	**MIMAT0027496**
36		MIMAT0004602	MIMAT0000725
37		MIMAT0015072	MIMAT0019882
38		MIMAT0028211	MIMAT0027670
39		MIMAT0012735	MIMAT0023713
40		MIMAT0000510	MIMAT0018444
41		MIMAT0004982	MIMAT0018925
42		MIMAT0027542	**MIMAT0005922**
43		MIMAT0015079	MIMAT0022947
44		MIMAT0027433	MIMAT0027389
45		MIMAT0027488	MIMAT0027542
46		MIMAT0018925	MIMAT0027510
47		MIMAT0027497	**MIMAT0004952**
48		MIMAT0007889	MIMAT0027430
49		MIMAT0019778	MIMAT0003298
50		MIMAT0019711	MIMAT0019883
51		MIMAT0000690	**MIMAT0028117**
52			MIMAT0028126
53			MIMAT0005573
54			MIMAT0021079
55			MIMAT0026486
56			MIMAT0005898
57			MIMAT0019776
58			MIMAT0027582
59			MIMAT0027488
60			MIMAT0019711
61			MIMAT0018094
62			MIMAT0007347
63			**MIMAT0005929**
64			MIMAT0030998
65			MIMAT0027563
66			MIMAT0027472
67			MIMAT0027426
68			MIMAT0031095
69			MIMAT0005934
70			MIMAT0005867
71			MIMAT0027550
72			MIMAT0005793
73			MIMAT0027390
74			MIMAT0004561
75			MIMAT0014998
76			MIMAT0018111
77			MIMAT0003885
78			MIMAT0000278
79			MIMAT0027433
80			MIMAT0004922
81			MIMAT0027359
82			MIMAT0027421
83			MIMAT0004982
84			MIMAT0022727
85			MIMAT0019903
86			MIMAT0018095
87			MIMAT0005901
88			MIMAT0030996
89			MIMAT0030420
90			MIMAT0004951
91			MIMAT0004687
92			MIMAT0004284
93			MIMAT0003288
94			MIMAT0006764
95			MIMAT0004948
96			MIMAT0019855
97			MIMAT0027575
98			MIMAT0019774
99			MIMAT0012735
100			MIMAT0022967
101			MIMAT0027596
102			MIMAT0027470
103			MIMAT0027511
104			MIMAT0004595
105			MIMAT0003308
106			MIMAT0000071
107			MIMAT0005914
108			MIMAT0027532
109			MIMAT0019816
110			MIMAT0003254
111			MIMAT0024598
112			MIMAT0004983
113			MIMAT0004602
114			MIMAT0019968
115			MIMAT0022691
116			MIMAT0019203
117			MIMAT0027650
118			MIMAT0028211
119			MIMAT0018078
120			MIMAT0004957
121			MIMAT0007889
122			MIMAT0018104
123			MIMAT0000440
124			MIMAT0003287
125			MIMAT0004792
126			MIMAT0019211
127			MIMAT0003240
128			MIMAT0023694
129			MIMAT0016880
130			MIMAT0019932
131			MIMAT0018198
132			MIMAT0027497
133			MIMAT0026621
134			MIMAT0015041
135			MIMAT0015030

Bold entries indicate miRNAs selected by all three methods

## Code availability

The custom R scripts used to generate the simulated datasets, implement the variable selection methods, and perform the analyses are available from the corresponding author upon reasonable request.

## References

[1] Jie C, Jiawei L, Shulin W, Sheng Y. Feature selection in machine learning: A new perspective. Neurocomputing. 2018;300:70–9. 10.1016/j.neucom.2017.11.077.

[2] Wu C, et al. A selective review of multi-level omics data integration using variable selection. High-Throughput. 2019;8(1):4. 10.3390/ht8010004. 30669303 PMC6473252

[3] Saeys Y, Inza I, Larrañaga P. A review of feature selection techniques in bioinformatics. Bioinformatics. 2007;23(19):2507–15. 10.1093/bioinformatics/btm344. 17720704

[4] Guyon I, Elisseeff A. An introduction to variable and feature selection. J Mach Learn Res. 2003;3:1157–82.

[5] Pudjihartono N, Fadason T, Kempa-Liehr AW, O’Sullivan JM. A review of feature selection methods for machine learning-based disease risk prediction. Front Bioinforma. 2022;2:927312. 10.3389/fbinf.2022.927312. PMC9580915 36304293

[6] Bolón-Canedo V, Sánchez-Maroño N, Alonso-Betanzos A, Benítez JM, Herrera F. A review of microarray datasets and applied feature selection methods. Inf Sci. 2014;282:111–35. 10.1016/j.ins.2014.05.042.

[7] Guyon I, Gunn S, Nikravesh M, Zadeh LA, editors. Feature Extraction: Foundations and Applications. Berlin: Springer; 2006.

[8] Tibshirani R. Regression shrinkage and selection via the LASSO. J Roy Stat Soc B. 1996;58(1):267–88. 10.1111/j.2517-6161.1996.tb02080.x.

[9] Bühlmann P, van de Geer S. Statistics for High-Dimensional Data. Berlin: Springer; 2011.

[10] Zou H, Hastie T. Regularization and variable selection via the Elastic Net. J Roy Stat Soc B. 2005;67(2):301–20. 10.1111/j.1467-9868.2005.00503.x.

[11] Freijeiro-González L, Febrero-Bande M, González-Manteiga W. A critical review of LASSO and its derivatives for variable selection under dependence among covariates. Int Stat Rev. 2022;90(1):118–45. 10.1111/insr.12469.

[12] Kursa MB, Jankowski A, Rudnicki WR. Boruta - A system for feature selection. Fund Inform. 2010;101(4):271–85. 10.3233/FI-2010-288.

[13] Degenhardt F, Seifert S, Szymczak S. Evaluation of variable selection methods for random forests and omics data sets. Brief Bioinform. 2019;20(2):492–503. 10.1093/bib/bbx124. 29045534 PMC6433899

[14] Acharjee A, Larkman J, Xu Y, Cardoso VR, Gkoutos GV. A random forest based biomarker discovery and power analysis framework for diagnostics research. BMC Med Genomics. 2020;13:178. 10.1186/s12920-020-00826-6. 33228632 PMC7685541

[15] Speiser JL, Miller ME, Tooze J, Ip E. A comparison of random forest variable selection methods for classification prediction modeling. Expert Syst Appl. 2019;134:93–101. 10.1016/j.eswa.2019.05.028. 32968335 PMC7508310

[16] Yokoi A, et al. Integrated extracellular microRNA profiling for ovarian cancer screening. Nat Commun. 2018;9:4319. 10.1038/s41467-018-06434-4. 30333487 PMC6192980

[17] Hosmer JDW, Lemeshow S, Sturdivant RX. Applied Logistic Regression. 3rd ed. Hoboken: Wiley; 2013.

[18] Steyerberg EW. Statistical Models for Prediction. In: Clinical Prediction Models. Statistics for Biology and Health. Cham: Springer; 2019. p. 25–61. 10.1007/978-3-030-16399-0_4.

[19] Friedman J, Hastie T, Tibshirani R, Narasimhan B, Tay K, Simon N. glmnet: Lasso and Elastic-Net Regularized Generalized Linear Models. 2025. R package version 4.1-9. https://CRAN.R-project.org/package=glmnet.

[20] R Core Team. R: A Language and Environment for Statistical Computing. Vienna, Austria; 2024. https://www.R-project.org/.

[21] Kursa MB, Rudnicki WR. Feature selection with the Boruta package. J Stat Softw. 2010;36(11):1–13. 10.18637/jss.v036.i11.

[22] Lee YS, Dutta A. MicroRNAs in cancer. Annu Rev Pathol. 2009;4:199–227. 10.1146/annurev.pathol.4.110807.092222. 18817506 PMC2769253

[23] Pichiorri F, De Luca L, Aqeelan RI. MicroRNAs: New players in multiple myeloma. Front Genet. 2011;2:22. 10.3389/fgene.2011.00022. 22303318 PMC3268577

